# Application of the TDR Sensor and the Parameters of Injection Irrigation for the Estimation of Soil Evaporation Intensity

**DOI:** 10.3390/s21072309

**Published:** 2021-03-25

**Authors:** Amadeusz Walczak, Mateusz Lipiński, Grzegorz Janik

**Affiliations:** Institute of Environmental Protection and Development, Wrocław University of Environmental and Life Sciences, pl. Grunwaldzki 24, 50-363 Wrocław, Poland; lipinski.mateusz1@gmail.com (M.L.); grzegorz.janik@upwr.edu.pl (G.J.)

**Keywords:** soil evaporation, mobile injection irrigation, empirical model, time-domain reflectometry

## Abstract

The objective of the study was to develop a precise method of determination of the evaporation rate in a soil irrigated with the use of a mobile injection irrigation system. Two methods of constructing functions approximating the value of evaporation have been developed. In the first method, the domain comprises the parameters of injection irrigation, i.e., the dose and the depth of injection, and in the second, the volumetric moisture of soil in the layer immediately below the soil surface, which was measured with time-domain reflectometry (TDR) sensors. For that purpose, a laboratory experiment was carried out, based on 12 physical models. The study was conducted on a natural soil material, with particle size distribution of its mineral parts corresponding to that of a loamy sand soil. It was demonstrated that evaporation intensity increases with irrigation and decreases with increase in the depth of water application. Using TDR sensors, it was also shown that evaporation intensity increases proportionally to the weighted arithmetic mean of the volumetric moisture. Comparison of the two methods indicates that the evaporation intensity of injection-irrigated soil can be estimated with higher accuracy when the domain of the approximating function is the injection depth and dose than when the domain of the function is the weighted mean of volumetric moisture of the surface horizon of the soil. However, the method using TDR sensors for the estimation of evaporation intensity of an injection-irrigated soil has a greater potential for the construction of universal approximating models. In addition, the advantage of the method based on the use of TDR sensors is that it uses arguments for the approximating function, $f_{2}\left(\tilde{\theta }\right)$, in real time.

## 1. Introduction

One of the most important natural resources is fresh water, access to which around the world is increasingly non-uniform and limited, both in time and in space [1,2]. The greatest amount of water, as much as 1300 km^3^, is used in agriculture. For comparison, water use in the industry amounts to approximately 400 km^3^, and the amount of water used for sanitary and household needs is 300 km^3^. In the sector of plant production, water is used mainly for irrigation [3]. This emphasises the need to apply irrigation methods that allow minimisation of losses of water. One of the primary factors affecting the level of such losses is the process of transition of the state of water from the liquid to the gaseous state on the open surface of soil, i.e., evaporation [4,5].

Evaporation intensity depends on type of soil and on the content of water in its surface horizon [6]. For example, Denisov et al. [7] developed a model of the intensity of the process of evaporation from bare soil. Simulations revealed that in windy conditions (5 m·s^−1^) and at soil saturation of 0.2 cm^3^·cm^−3^, the intensity of evaporation was approximately 3.5 mm·day^−1^, and at soil saturation of 0.5 cm^3^·cm^−3^, as much as 5.5 mm·day^−1^. In another study [8], it was demonstrated that when the volumetric moisture of the surface horizon of soil was 0.03 cm^3^·cm^−3^, the intensity of evaporation was close to zero, and at moisture of 0.05 cm^3^·cm^−3^, it was as much as 0.8 mm·day^−1^. The intensity of water evaporation also differs for various types of soil [9]. In sandy soils, at soil saturation of 0.25 (−), evaporation is close to the maximum value, while in the same conditions, in a loamy soil, it is close to zero.

Evaporation intensity is also affected by physiographic factors, such as, e.g., the kind of surface cover. Various organic and inorganic mulches are used in agriculture, causing a reduction of evaporation [10,11,12]. For example, in the case of using a mulch of spruce tree, the reduction of evaporation relative to bare soil is 50%, and when mulching with an inorganic material is applied, the reduction is as much as 75%. The thickness of the mulch layer does not have any significant impact [13]. The application of a 5 centimetre layer of mulch resulted in moisture reduction by approximately 60%, while in the case of a 15 centimetre layer, the reduction amounted to 70%. The topography of the area is also an important factor for the intensity of evaporation [14,15].

The intensity of the process of water evaporation from soil also depends on the meteorological conditions [16,17]. This is related to the fact that the process of evaporation takes place at the boundary between soil and atmosphere. Relative humidity at the soil surface is the most important element, affecting the intensity of water evaporation. It is a measure of the availability of water vapour, and depends, among other things, on wind direction and velocity [18,19,20]. The status of the atmosphere is also described by the influx of short-wave radiation and long-wave radiation from the atmosphere [21], air temperature, atmospheric pressure, and wind. In the study by Denisov et al. [7] mentioned above, the authors demonstrated that under zero-wind conditions, the intensity of evaporation amounts to approximately 1–2 mm·day^−1^, with soil saturation with water in the range of 0.2–1.0 cm^3^·cm^−3^. For the same moisture conditions in the surface soil horizon, but with wind velocity of 5 m·s^−1^, evaporation intensity is in the range of 4.0–6.0 mm·day^−1^, and with wind velocity of 11 m·s^−1^, the evaporation is as high as 10 mm·day^−1^. This maximum value, however, appears when the soil is in the state of full saturation with water. Similar conclusions were formulated in another study [22], where the authors additionally proved that the higher the temperature of the incoming mass of air, the more intense the process of evaporation.

Contemporary methods for the determination of evaporation intensity can be classified in 4 groups: lysimetric measurements, models using satellite imaging, (semi)empirical mathematical formulae, and phenomenological models [16].

Evaporation intensity determined by means of measurements in lysimeters is used primarily for calibration of mathematical models [23,24]. A lysimeter has the form of a metal container inserted into the ground. Measurement of evaporation intensity consists in determinations of the weight of the container, which changes in the course of the day due to, among other factors, water evaporation from the soil [25]. The advantage of this method is that the measurements are conducted on samples with undisturbed structure, and that there is the possibility of analysing the water balance of the soil monolith [26,27].

Satellite imaging, data from which are used for models, provided important progress in studies on evaporation intensity of larger areas [28,29,30,31]. As an example, the GLEAM model (Global Land surface Evaporation: the Amsterdam Methodology) uses data from eight different sources (satellites) which provide information on radiation, precipitation, surface moisture and temperature of soil, air temperature, depth of vegetation, or snow cover [32]. On the basis of such information and with the use of an evaporation equation [33], the GLEAM model generates data on evaporation intensity at a global scale with a correlation coefficient of 0.8. The GLEAM project is constantly updated. Currently, work on version 3 has been completed [34]. This update includes a broader range of input data (including data from the Soil Moisture and Ocean Salinity—SMOS satellite), and permits the analysis of a larger number of water balance components (soil moisture in the root zone) [35].

The third group of methods is that of empirical mathematical formulae. Those formulae require the determination of a large amount of input data [36]. It needs to be emphasised that the empirical models are usually dedicated to specific regional, climatic, and soil conditions [37], which is their main shortcoming [38]. The most frequently used formula is the Penman equation [39]. The input data in that formula include the values of air humidity deficit and wind velocity. That formula has been the basis for the development of numerous modifications, including the frequently used FAO-56 model [16,40,41].

Still, another possibility of describing the intensity of the process of evaporation is provided by phenomenological models, i.e., in which the process is described by means of relations between selected physical values. The relations are expressed by means of mathematical formulae. Due to the complexity of the phenomenon of evaporation, it is necessary to introduce a large number of input data which characterise the soil medium and provide information on the changing atmospheric conditions. For example, in References [22,42] and, the authors analysed the effect of initial and boundary conditions of the same model based on the Navier-Stokes equations and on Darcy’s Law. The analysed variables were the following: wind velocity and the parameters of the inflowing air masses, hydraulic parameters of the ground, and heat transfer in a porous medium. According to Reference [17], the rate of evaporation can be determined by dividing the process into two phases: the intense process of evaporation augmented by capillary rise, and the insignificant process of water vapour diffusion from soil.

The method of mobile injection irrigation [43] is an innovative concept of a system which allows to minimise evaporation. With this method of irrigation, water is applied directly to the rhizosphere by means of injectors. The application of a water dose for an individual plant takes several seconds, due to which the pressure is high and amounts to approximately 4 bar. The injectors are installed on a mobile platform which can move over fields that need irrigation. The system of mobile injection irrigation is characterised by the fact that water can be applied below the soil surface, like in a subsurface drip line. However, in case of injection irrigation, the depths of injection can be adapted to places where the main root mass is actually situated. Therefore, this approach allows the minimisation of water losses resulting from evaporation. Another advantage of injection irrigation over subsurface drip lines is mobility. The installation of a drip line may turn out to be useful when the growing season is full of rainfall. On the other hand, it should be taken into account that the system can be used in vegetable cultivations such as celery, leek, cabbage, and sown cultures like, for example, carrot and parsley (with wide inter-rows). In a comprehensive evaluation of the usefulness of the concept of a mobile system of injection irrigation, one should also take into consideration the economic aspect, e.g., the fuel costs.

The conditions of evaporation during injection irrigation differ from those in the case of other irrigation systems. It should be emphasised that the aspect of determination of the rate of evaporation has not been an object of research so far for the conditions of injection irrigation, even though it is an element that determines the volume of water used in plant production.

In view of the above, the objective of the study was to develop a precise method for assessing the intensity of evaporation from a soil irrigated by means of the mobile system of injection irrigation. For doing this, methods have been developed for the construction of two functions approximating the values of evaporation. In the first of those methods, the domain is the dose and depth of water injection, and in the second, the volumetric moisture of soil in the layer immediately below the surface.

## 2. Material and Methods

**Figure 1:**
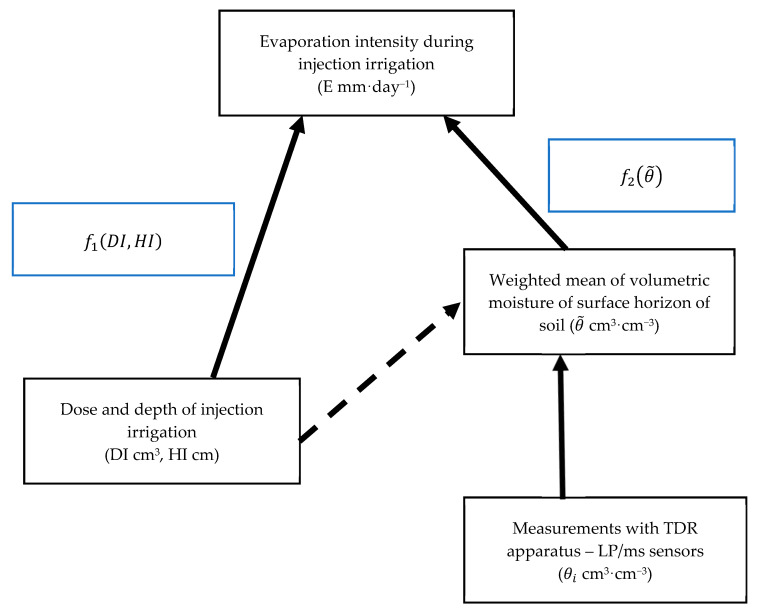
Input data for functions approximating evaporation intensity in the conditions of injection irrigation.

The experiments were conducted under controlled conditions, at the Laboratory of Soil Physics and Modelling of Environmental Processes, Institute of Environmental Protection and Development, Wrocław University of Environmental and Life Sciences (Wrocław, Poland). This allowed conducting the experiments in such a way that the intensity of evaporation from soil surface depended only on the dose and depth of water injection, i.e., the parameters which determine the distribution of volumetric moisture immediately beneath the soil surface. Other factors affecting the process during the experiment were constant. Figure 1 presents a schematic of the concept of the present study. It indicates that evaporation can be estimated on the basis of injection parameters, i.e., the dose (DI) and depth (HI) ($f_{1}\left(DI,HI\right)$), or on the basis of the weighted mean of volumetric moisture, $\left(\tilde{\theta }\right)$ ($f_{2}\left(\tilde{\theta }\right)$), determined on the basis of point-wise measurements with a TDR (time-domain reflectometry) apparatus equipped with LP/ms (laboratory probe/moisture salinity) sensors. The probes were designed and manufactured at the Institute of Agrophysics PAS in Lublin (Poland) (Available online: https://www.e-test.eu/) (accessed on 24 April 2021) [44]. Due to their small dimensions (body length and diameter of 5.0 and 0.8 cm respectively, rod length and diameter of 5.3 cm and 0.8 mm), the sensors are used primarily in laboratory experiments. The method of determination of the volumetric moisture of soil consists in fully automatic measurement of the relative permittivity of a porous medium. Figure 1 indicates that the weighted mean of volumetric moisture depends on the dose and depth of injection, although this aspect is not analysed in the study.

$f_{1}\left(DI,HI\right)$—function approximating evaporation intensity on the basis of parameters of injection irrigation,
$f_{2}\left(\tilde{\theta }\right)$—function approximating evaporation intensity on the basis of weighted mean of volumetric moisture of surface horizon of soil,
DI—dose of water applied during injection irrigation (cm^3^),
*HI*—depth of injection (cm).

### 2.1. Experiment

**Figure 2:**
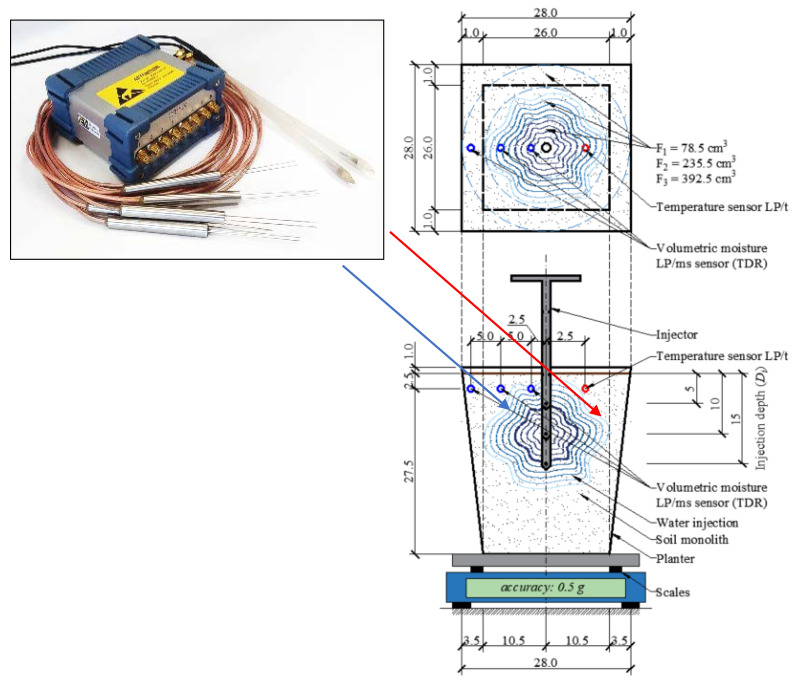
Schematic of a single pot, with the injector, moisture, and temperature sensors, and the balance. F1, F2, and F3—representative areas for LP/ms (laboratory probe/moisture salinity) probes, dimensions are displayed in centimetres.

The experiment aimed at the construction of functions $f_{1}\left(DI,HI\right)$ and $f_{2}\left({\tilde{\theta }}_{n}\right)$ was conducted on 12 identical physical models (pots with a volume of 17 dm^3^). The upper surface of the pots was uncovered, and all the walls were impermeable. In each of the pots, at the depth of 2.5 cm, 3 TDR sensors (LP/ms) were installed horizontally, for the measurement of volumetric moisture, and additionally, at the same depth, a temperature sensor (LP/t) was installed [45,46,47]. The positioning of the sensors in the physical model and dimensions of the pots are presented in Figure 2. For the measurement of the loss of pot mass due to evaporation, an electronic balance was used, Radwag PM 50.C32 (manufacturer RADWAG Wagi Elektroniczne Witold Lewandowski, Poland) [48]. The balance allows readings in the range of up to 60 kg with 0.5 g accuracy [49,50].

The natural soil material building each of the physical models was a porous structure, collected from the top 30 cm layer of soil, from a field situated within the area of the Vegetable and Ornamental Plants Research and Didactics Station in Psary (Wrocław, Poland) (51°19′ N, 17°30′ E). The volume of the natural soil material was sufficient to fill at least 15 pots. Soil is a porous medium with a strong diversification of structure, even for points situated at small distances from one another [51]. Therefore, to improve the uniformity of conditions in each of the pots, organic parts and the fraction of particles larger than 2 mm were removed from the natural disturbed soil material, as they have a detrimental effect on the correctness of reading of LP/ms probes. The natural material prepared in this manner was compacted in the pots, obtaining a disturbed, natural porous medium, for which the particle size distribution of its mineral parts, according to the USDA (Universal Soil Classification System) classification, corresponded to that of a loamy sand [52]. The bulk density of the material was 1.26 g·cm^−3^. The natural porous material used for the building of the physical model differs from soil in its natural state only in not having any content of organic parts and of the fraction of particles larger than 2 mm. In that case, the difference also involved the change of soil structure and compaction. Determinations of mineral particles were made on the basis of sieve analysis and areometric measurements [53]. The results of the analysis showed that the clay fraction (particle size lower than 0.002 mm) was present in 1% of the soil material, the silt fraction (0.002–0.050 mm) covered 12% of the soil material, and the sand fraction (0.050–2.000 mm) was in 87% of the soil material.

**Figure 3:**
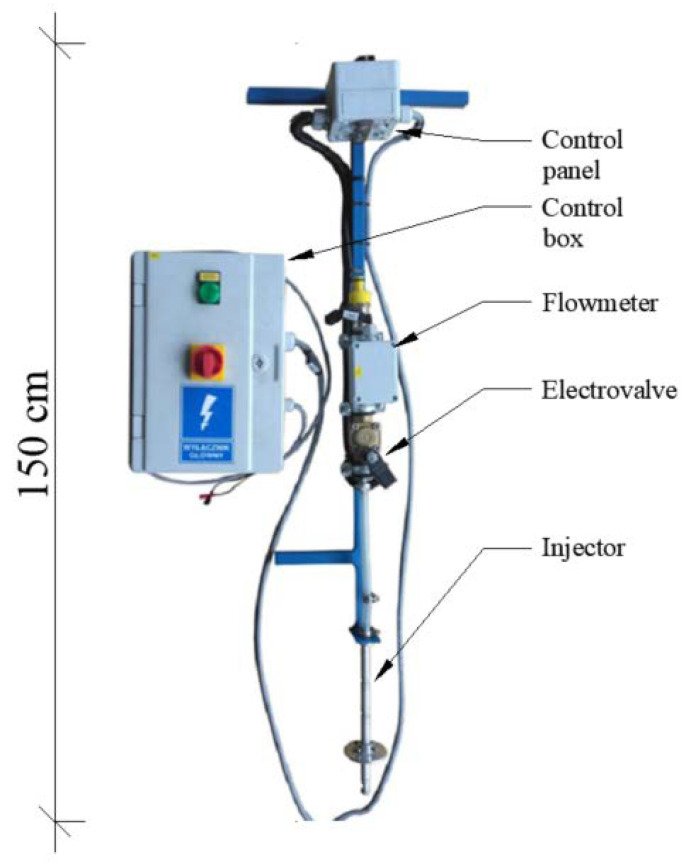
Prototype injector for precision application of water into the physical model at desired depth [54].

For the injection of a precisely determined dose of water into the interior of the pot, at a desired depth, a prototype injector was used (Figure 3). The injector was designed within the framework of project BIOSTRATEG3/343547/NCBR/2017 by employees of the Department of Plastic Forming and Metrology, Wrocław University of Engineering (Poland) [54]. The most important element of the injector is a 20 cm stainless-steel pin, extending from the metal body of the device. At the tip of the pin, there is an exchangeable conical nozzle with a side outlet aperture through which a precise amount of water is ejected under the pressure of 4 bar. In addition, the metal structure of the injector mounts a control panel, which allows the selection of the irrigation dose, a flow meter, and an electrovalve (solenoid valve).

Series of experiments were conducted to determine the functions approximating evaporation during injection irrigation. In each of those series, a sample was irrigated once using the injector. The injection doses were 250, 450, 750, or 1000 cm^3^. For each of the doses, injection was performed by applying the water at depths of 5, 10, or 15 cm. During each of those series, with a 1 min time-step, volumetric moisture was recorded at 3 points using TDR sensors, type LP/ms, and soil temperature at one point. Each of the series lasted for 40 days. During that period, starting from the moment of water injection, each of the pots was weighed once a day, at midday. During the experiment, the temperature in the laboratory varied from 21.5 to 29.1 °C (average 24.1 °C), while the relative humidity varied from 53% to 92% (average 73%).

Next, an identical experiment was conducted, but in only 3 pots. Its purpose was the estimation of the goodness of the functions $f_{1}\left(DI,HI\right)$ and $f_{2}\left({\tilde{\theta }}_{n}\right)$. In that experiment, the pots were filled with a fresh batch of the same natural soil material as in the first experimental series. The additional experiment simulated the injection of 550 cm^3^ of water to the depths of 5, 10, and 15 cm.

### 2.2. Calculations

Due to the fact that in the conditions of the experiment the change of pot weight was caused solely by evaporation from the soil surface, on each day, the intensity of evaporation in the conditions of injection irrigation could be calculated from the following formula:

(1) $$E_{n}=10\cdot \left(m_{n}-m_{n+1}\right)\cdot \rho_{w}^{-1}\cdot F^{-1}\cdot \Delta T^{-1}$$

where: $E_{n}$—evaporation intensity on the n-th day (mm·day^−1^), $m_{n},\;\left(m_{n+1}\right)$—mass of the pot on the n-th day (day n + 1) (g), $\rho_{w}$—water density (g·cm^−3^), *F*—upper surface area of the pot (cm^2^), ΔT—time step (day).

On the basis of information on the weight of each of the pots on the consecutive days of the experiment, one can also calculate the total evaporation in the period from the initial moment (water injection) to a current point in time. The total evaporation was denoted with symbol $E_{1-n}$ and expressed as:

(2) $$E_{1-n}=10\cdot \left(m_{1}-m_{n}\right)\cdot \rho_{w}^{-1}\cdot F^{-1}$$

where: $E_{1-n}$—value of evaporation from the initial moment (1st day) to a current moment (n-th day), determined by the change of the pot weight (mm), $m_{1}$ ($m_{n}$)—pot mass on the 1st (n-th) day (g), and other symbols as in Equation (1).

Subsequently, on the basis of the results of the measurements taken with identical LP/ms probes, the mean weighted volumetric moisture of the surface horizon of the soil was calculated as follows:

(3) $${\tilde{\theta }}_{n}=\;\frac{\theta_{n}^{1}\cdot F_{1}+\theta_{n}^{2}\cdot F_{2}+\theta_{n}^{3}\cdot F_{3}}{F_{1}+F_{2}+F_{3}}$$

where: ${\tilde{\theta }}_{n}$—weighted mean of volumetric moisture of surface horizon of the soil (cm^3^·cm^−^^3^), $\theta_{n}^{1},\;\theta_{n}^{2},\;\theta_{n}^{3}$—maximum volumetric moisture recorded on the n-th day by sensors distant by 2.5 cm ($\theta_{n}^{1}$), 7.5 cm ($\theta_{n}^{2}$), and 12.5 cm ($\theta_{n}^{3}$) from the point of injection (cm^3^·cm^−^^3^), $F_{1},\;F_{2},\;F_{3}$—areas representative for each of the LP/ms sensors (Figure 2), of 78.5, 235.5, and 392.5 cm^2^, respectively.

The differences in the size of the representative areas result from the positioning of the LP/ms sensors, as presented in Figure 2. On the basis of the individual 24 h weighted mean values of volumetric moisture, ${\tilde{\theta }}_{n},$ one can also calculate the arithmetic mean of moisture in the period from the initial moment (1st day) to the current point in time (n-th day). This mean value was denoted with the symbol ${\tilde{\theta }}_{1-n}$ and expressed by means of the formula:

(4) $${\tilde{\theta }}_{1-n}=\;\frac{1}{n}\overset{n}{\underset{i=1}{\sum }}{\bar{\theta }}_{i},\;$$

where: ${\tilde{\theta }}_{1-n}$—arithmetic mean value of the weighted mean of volumetric moisture from the initial moment (1st day) to the current day (n-th day) (cm^3^·cm^−^^3^), n—number of measurement days (−), and other symbols as in Equation (3).

In the literature, the accuracy of approximation of a function is often given as a statistical value determined on the basis of data unknown to the model. The data can be, e.g., the mean value, the median, or the relative mean square error (RMSE) between the predicted and the actual values [55]. In this study, for the set of data acquired on the basis of the above measurements made on the physical models and on the basis of calculations allowing the determination of the value of $E_{1-n}$ (value of evaporation from the initial moment (1st day) to a current moment (n-th day)) permitted the development of two approximating functions. In the first function ($E_{1-n}^{f_{1}}=f_{1}\left(DI,HI\right)$), the domain is the dose of water injection and the depth of its application, and in the second function ($E_{1-n}^{f_{2}}=f_{2}\left(\tilde{\theta }\right)$), the domain is the weighted mean of volumetric moisture of the surface soil horizon, calculated on the basis of measurements with the TDR equipment. The values of functions $f_{1}$ and $f_{2}$ are the values of evaporation from the initial moment (1st day) to a current day (n-th day). The measure of fit of the functions to the results of measurements (first experiment—12 pots) of the value of $E_{1-n}$ was the mean square from the differences, calculated in accordance with the formula:

(5) $$S_{f_{1},\left(f_{2}\right)}^{1}=\frac{1}{m}\overset{m}{\underset{j=1}{\sum }}{\left(E_{1-n,j}-E_{1-n,j}^{f_{1},(f_{2})}\right)}^{2},\;$$

where: $S_{f_{1},\left(f_{2}\right)}^{1}$—mean square from differences between values $E_{1-n,j}$ and $E_{1-n,j}^{f_{1},(f_{2})}$ (mm^2^), *m*—number of compared pairs: 12 (−), $E_{1-n,j}^{f_{1},(f_{2})}$—value of evaporation from the initial moment (1st day) to a current day (n-th day) from the approximating functions *f*_1_ or *f*_2_ (mm), and other symbols as in Equation (2).

To evaluate the suitability of the functions $f_{1}\left(DI,HI\right)$ and $f_{2}\left(\tilde{\theta }\right)$ for the estimation of the value of evaporation during injection irrigation, the measures of the goodness of approximation ($S_{f_{1},\left(f_{2}\right)}^{2}$) were calculated. It was assumed that they should be the means from the modules of differences of the values of evaporation obtained on the basis of a separate experiment (second experiment—3 pots) and of values obtained from the approximating functions. The separate experiment was conducted in three replicates, in the same manner, in identical pots filled with an identical natural soil material, and in the same environmental conditions as the experiment presented in Figure 2. The measures of goodness were calculated using the following formula:

(6) $$S_{f_{1},\left(f_{2}\right)}^{2}=\frac{1}{m}\overset{m}{\underset{j=1}{\sum }}\left|E_{1-n,j}-E_{1-n,j}^{f_{1},(f_{2})}\right|$$

where: $S_{f_{1},\left(f_{2}\right)}^{2}$—average of modules from differences between values of $E_{1-n,j}$ and $E_{1-n,j}^{f_{1},(f_{2})}$ (mm), and other symbols as in Equation (5).

## 3. Results and Discussion

**Figure 4:**
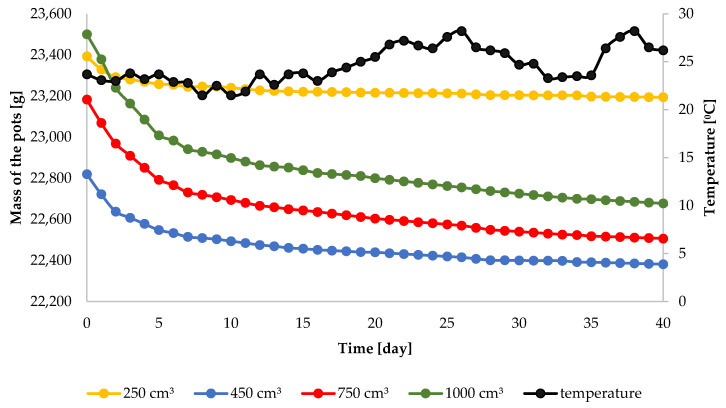
Dynamics of pot mass during 40 days after the injection of 250, 450, 750 and 1000 cm^3^ of water at the depth of 5 cm.

The diurnal patterns of mass and temperature of a pot, injection-irrigated with water dose of 250 cm^3^ at the depth of 5 cm, are presented in Figure 4. Over the entire period of the experiment, the mass of the pot decreased: on the first day it was 23,392.9 g and on the final day it was lower by 199.5 g, at 23,193.4 g. On the initial days of the experiment, the decrease of pot mass was distinctly greater than on the final days. For instance, on the second day, the mass decrease was 37.6 g, and on day 14 it was as little as 2.1 g. Analogous regularities can be observed for the other water doses. The mean diurnal temperature at the depth of 2.5 cm showed a variation, in the range from 21.5–28.2 °C. Nevertheless, in the course of the experiment, the diurnal variation of temperature was the same for every pot. Therefore, the temperature was not a factor differentiating evaporation. As mentioned earlier, the decrease of pot mass was caused solely by evaporation. Therefore, on the basis of the data given in Figure 4, one can calculate the value of the diurnal intensity of evaporation for each water dose and injection depth. Similar results, though not for the case of injection irrigation, were obtained in a study by Lejcuś et al. [50]. In that study, the authors monitored the loss of mass of a pre-wetted soil monolith with dimensions similar to those of the pot in Figure 2. After 10 days, the loss amounted to approximately 400 cm^3^.

### 3.1. Evaporation Intensity in Relation to Injection Depth and Dose ($f_{1}\left(DI,HI\right)$)

**Figure 5:**
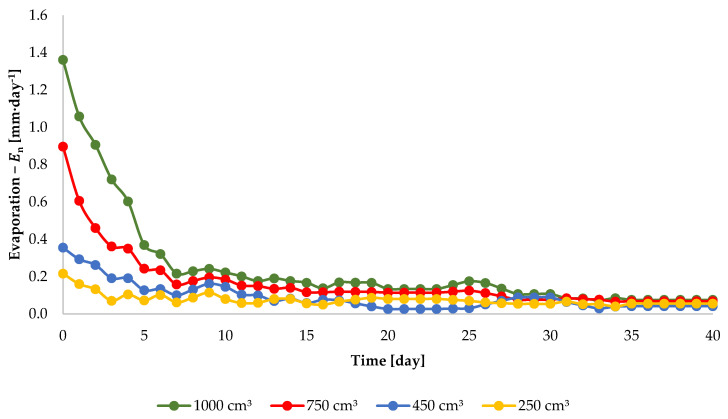
Diurnal evaporation intensity, $E_{n}$, after the injection of 250, 450, 750 and 1000 cm^3^ of water at the depth of 10 cm.

The values of diurnal evaporation intensity for injection irrigation with water doses of 250, 450, 750, and 1000 cm^3^ at the injection depth of 10 cm (Equation (1)) are presented in Figure 5. The values of the diurnal evaporation intensity increase with injection doses. For instance, on day 5 of the experiment, with injection dose of 250 cm^3^, evaporation rate was 0.10 mm·day^−1^, and for doses of 450, 750 and 1000 cm^3^, it amounted to 0.19, 0.35, and 0.60 mm·day^−1^, respectively. This regularity can be observed until day 15 of the experiment. In subsequent periods, when the diurnal evaporation intensity was practically approaching zero (below 0.07 mm·day^−1^), the values of $E_{n}$ calculated from Equation (1) were unstable. This is due to the fact that the accuracy of the balance is 0.5 g. For this reason, deviations from the regularity are observed in those periods. In addition, the value of $E_{n}$ decreases with the time elapsed from the moment of injection. For instance, on day 2 from the moment of injection, for the dose of 750 cm^3^, the value of evaporation intensity was 0.61 mm·day^−1^, on day 6, 0.24 mm·day^−1^, and on day 14, as little as 0.14 mm·day^−1^. This regularity is observed for each of the injection doses.

**Figure 6:**
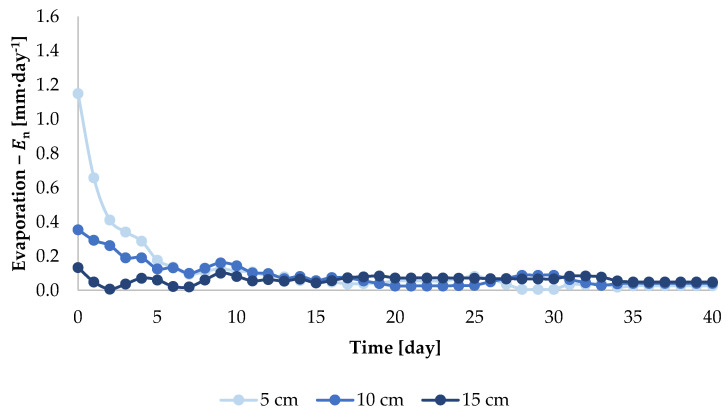
Evaporation intensity, $E_{n}$, after the injection dose of 450 cm^3^ at depths of 5, 10 and 15 cm.

The diurnal values of evaporation intensity calculated on the basis of Equation (1) for injection depths of 5, 10 and 15 cm, on the example of the dose of 450 cm^3^, are presented in Figure 6. It can be inferred from Figure 6 that the greater the depth of injection, the lower the value of $E_{n}$. For instance, on day 5 of the experiment, for an injection depth of 5 cm, the value of $E_{n}$ was 0.29 mm·day^−1^, for 10 cm was 0.19 mm·day^−1^, and for the depth of 15 cm, the value of $E_{n}$ was 0.07 mm·day^−1^. The above analyses indicate that evaporation intensity for injection irrigation increases with increasing doses and decreases with increasing depth of injection. This fact indicates the correctness of the experiment and of the calculations.

Due to the fact that values of diurnal intensity of evaporation (Figure 5 and Figure 6) were lower than 0.2 mm·day^−^^1^ since day 15 of the experiment, the calculations of the value of $E_{1-n}$ (Equation (2)) for each dose and for each injection depth were made for n = 15 days. The results of the calculation of the value of $E_{1-15}$ are presented in Table 1.

Data in Table 1 confirmed that evaporation calculated for the period from the moment of injection until day 15 ($E_{1-15}$) increases with increasing dose and decreases with increasing depth of injection. The highest value of $E_{1-15}$, of 7.68 mm, was obtained for the dose of 1000 cm^3^ and injection depth of 5 cm, and the lowest, 0.64 mm, for the dose of 250 cm^3^ and injection depth of 15 cm.

To find a suitable function expressing the values of $E_{1-15}$, the domain of which is the injection dose and depth, the suitability of various classes of functions was analysed. In fact, the empirical data were approximated with a modified logistic function [56,57]. The function from this class correctly described the relations in which the values increase with the increase of one term and decrease with the increase of another term [58,59]. The function has the following form:

(7) $$E_{1-15}^{f_{1}}\left(DI,HI\right)=\frac{10\cdot A}{1+B\cdot e^{-C\;\cdot \;\left(\left(DI^{max}-DI\right)\cdot 10^{-3}\;\cdot \;HI\right)}},$$

where: $E_{1-15}^{f_{1}}\left(DI,HI\right)$—approximated function *f_1_* of evaporation for injection irrigation, from the initial moment (1st day) until day 15 (mm), DI—dose of water applied during injection irrigation (cm^3^), *HI*—injection depth (cm), *A* (mm), *B* (−), *C* (cm^−4^)—empirical coefficients.

The values of parameters *A*, *B*, and *C* were chosen so that the sum of squares of differences, $S_{f_{1},\left(f_{2}\right)}^{1}$ (Equation (5)), was the smallest. The optimisation procedure was conducted with the use of an algorithm developed in the Python environment [60,61]. Based on pilot calculations, the optimisation of the parameters was performed for all variants of A, B, C in the range of the set {−50.00, −49.99…, 49.99, 50.00}. Ultimately, the function $E_{1-15}^{f_{1}}\left(DI,HI\right)\;$ assumed the form:

(8) $$E_{1-15}^{f_{1}}\left(DI,HI\right)\;=\frac{10\cdot 7.9}{1+19.54\cdot e^{-0.44\cdot \left(\left(DI^{max}-DI\right)\cdot 10^{-3}\;\cdot HI\right)}}$$

where: all symbols are as in Equation (7).

**Figure 7:**
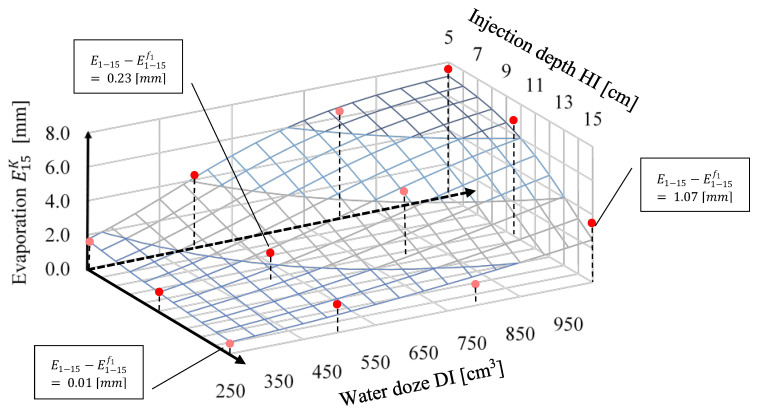
Function of evaporation (after 15 days) in relation to injection depth (HI cm) and water dose (DI cm^3^): $E_{1-15}^{f_{1}}\left(DI,HI\right)$.

In such a case of A, B, C, the value of the sum of squared differences, $S_{f_{1}}^{1}$ (Equation (5)), was 0.22 mm^2^. The shape of the function $E_{1-15}^{f_{1}}\left(DI,HI\right),$ with coefficient values *A* = 7.9 mm, *B* = 19.54, and *C* = 0.44 cm^−4^, is presented in Figure 7. Figure 7 also presents the values of $E_{1-15}$ obtained on the basis of measurements. Points with stronger red colour intensity are situated above surface $E_{1-15}^{f_{1}}\left(DI,HI\right)$, and lighter-coloured points are below that surface. The selected differences between $E_{1-15}$ and $E_{1-15}^{f_{1}}$ are also presented in Figure 7. Also, this figure shows that an increase of injection dose causes increased evaporation. For example, in the case of DI of 250 cm^3^ and HI of 10 cm, the value of evaporation calculated for the initial 15 days on the basis of the approximated function amounts to 1.05 mm ($E_{15}^{K}\left(250\;{\mathrm{cm}}^{3},10\;\mathrm{cm}\right)=1.05\;\mathrm{mm}$), while for DI of 1000 cm^3^ and HI of also 10 cm, the value of evaporation for the initial 15 days amounts to 6.37 mm ($E_{15}^{K}\left(250\;{\mathrm{cm}}^{3},10\;\mathrm{cm}\right)=6.37\;\mathrm{mm}$). In addition, we can observe that with an increase of HI depth, the values of the function $E_{1-15}^{f_{1}}\left(DI,HI\right)\;$ decrease. For example, for DI of 750 cm^3^ and HI of 5 cm, the value of evaporation for the initial 15 days amounts to 6.94 mm ($E_{15}^{K}\left(750\;{\mathrm{cm}}^{3},\;5\;\mathrm{cm}\right)=6.94\;\mathrm{mm}$), while for the same irrigation dose and HI of 15 cm, the value of evaporation for the initial 15 days amounts to 1.66 mm ($E_{15}^{K}\left(750\;{\mathrm{cm}}^{3},15\;\mathrm{cm}\right)=1.66\;\mathrm{mm}$).

### 3.2. Evaporation Intensity in Relation to the Volumetric Moisture of the Surface Horizon of Soil ($f_{2}\left(\tilde{\theta }\right)$)

**Figure 8:**
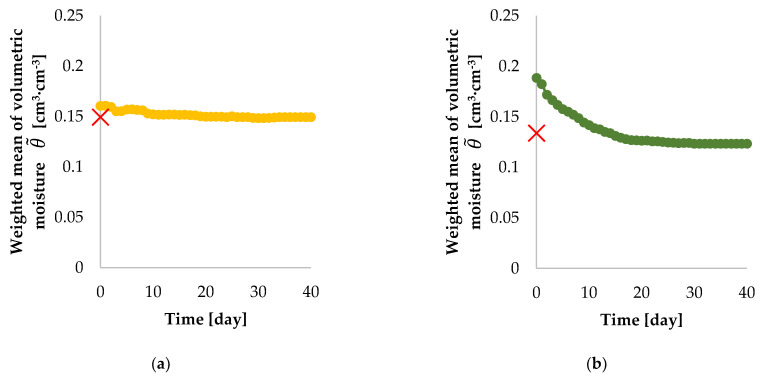
Dynamics of volumetric moisture of soil after the injection of 250 (**a**) and 1000 (**b**) cm^3^ of water at the depth of 10 cm.

Figure 8 presents the dynamics of moisture from the moment of injection of 250 and 1000 cm^3^ of water, for the example of the injection depth of 10 cm. For the dose of 250 cm^3^, the average moisture for 3 sensors immediately before the injection (red cross) was 0.15 cm^3^·cm^−3^, and for the dose of 1000 cm^3^, 0.13 cm^3^·cm^−3^. The corresponding values immediately after the injection were 0.16 and 0.19 cm^3^·cm^−3^, respectively. Therefore, for the dose of 250 cm^3^, the increase of moisture was 0.01 cm^3^·cm^−3^, and for the dose of 1000 cm^3^, was 0.05 cm^3^·cm^−3^. This indicates that the greater the dose, the higher the increase of volumetric moisture immediately after the injection. This regularity occurs for each of the depths of injection. In the course of the experiment, after the moment of injection, the values of volumetric moisture decreased with the time. The rate of the decrease increased with the injection dose. For the dose of 250 cm^3^, the decrease was 0.01 cm^3^·cm^−3^, and for the dose of 1000 cm^3^, it was 0.07 cm^3^·cm^−3^. This indicates that the greater the dose, the stronger the decrease of mean moisture.

**Figure 9:**
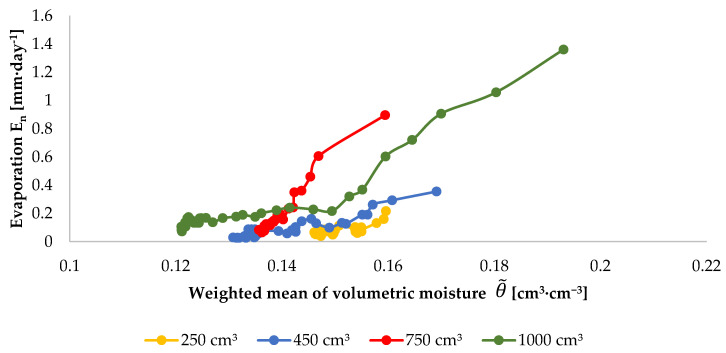
Evaporation versus weighted mean of volumetric moisture of soil after the injection of 250, 450, 750 and 1000 cm^3^ of water at the depth of 10 cm.

**Figure 10:**
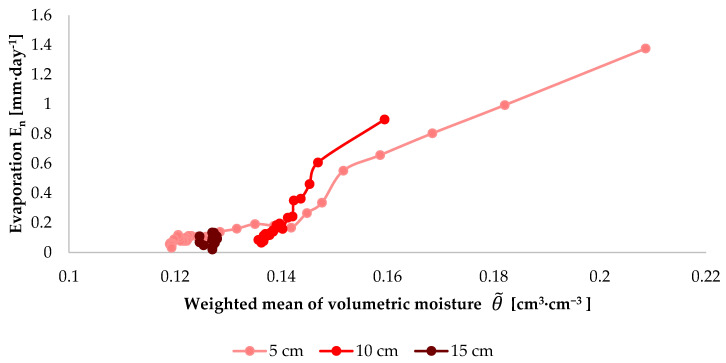
Evaporation versus weighted mean of volumetric moisture after the injection dose of 750 cm^3^ at depths of 5, 10, and 15 cm.

**Figure 11:**
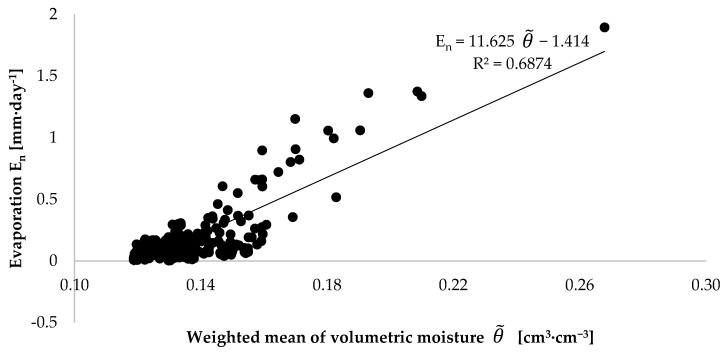
Relationship between daily evaporation and weighted mean of volumetric moisture for the 12 pots, calculated separately for each day on which the experiment was performed.

One of the factors determining the intensity of evaporation is the volumetric moisture of the top horizon of soil. This parameter constitutes the basis for the construction of boundary conditions in models concerning evaporation. For example, in the meta-analysis data-driven approach performed by Merlin et al., it constitutes the primary calibration factor [62]. In a study by Chanzy and Bruckler, volumetric moisture, next to wind, is the main variable which allows the description of the daily potential evaporation [63]. A correlation between evaporation and moisture was also noted in laboratory experiments involving small soil monoliths (several hundred cm^3^) [64,65]. To demonstrate the correctness of this observation, a graph was plotted, illustrating the relation between the diurnal evaporation intensity calculated from Equation (1) and the weighted mean of volumetric moisture in the surface horizon from Equation (3). For a preselected depth of 10 cm, for the example of the injection dose of 1000 cm^3^, the maximum value of evaporation intensity is 1.36 mm·day^−1^ at a weighted mean of volumetric moisture equal to 0.193 cm^3^·cm^−3^. For the same case, when the weighted mean of volumetric moisture is 0.122 cm^3^·cm^−3^, the diurnal evaporation intensity is as low as 0.11 mm·day^−1^ (Figure 9). This regularity is also observed for the remaining injection doses and depths (Figure 10). It should be noted that the higher the weighted mean of volumetric moisture, the higher the values of diurnal evaporation intensity. This relationship is presented in Figure 11.

**Figure 12:**
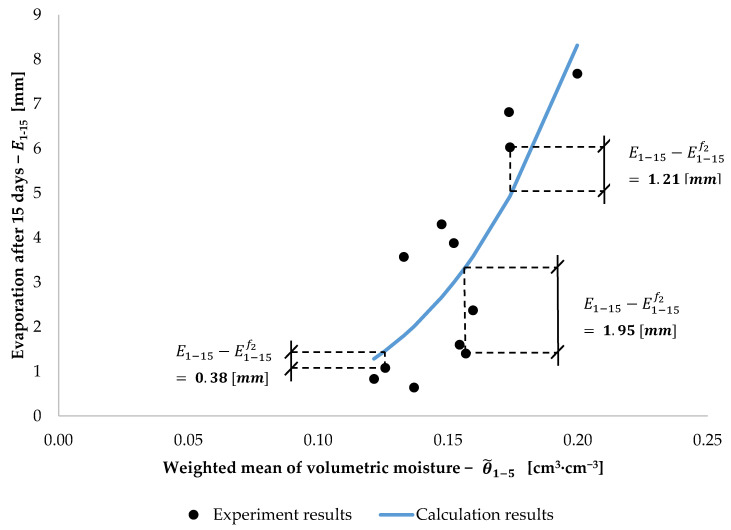
The value of 15-day evaporation, $E_{1-15}^{f_{2}}$, versus the weighted mean of volumetric moisture from the moment of injection (day 1) to day 5: ${\tilde{\theta }}_{1-5}$.

In this study, an attempt was also made at the construction of a function $f_{2}\left(\tilde{\theta }\right)$ which will approximate the same value as function $f_{1}\left(DI,HI\right)$, i.e., $E_{1-15}$, but on the basis of the weighted mean of volumetric moisture. The range of the analyses included fitting tests for the following functions: linear (y = a·x + b), exponential (y = a·x^b^), logarithmic (y = a·ln(x) + b and y = a·log_b_x), and a power function (y = a·e^b^^·^^x^). For those functions, sets consisting of pairs $E_{1-15}\left(HI,\;DI\right)$ and ${\tilde{\theta }}_{1-n}\left(HI,\;DI\right)$ were analysed for n (number of days from the moment of injection) from 1 to 15. The optimisation procedure for parameters a and b was performed in a manner similar to that for $E_{1-15}^{f_{1}}\left(DI,HI\right)$, i.e., with the use of an algorithm created in the Python environment. The best fit (the lowest values of the mean square from the differences: $S_{f_{2}}^{1}$) was obtained for the exponential function (y = a·x^b^) and when a = 3523.93 mm, b = 3.7559 (−), and the weighted mean was calculated for the period of 5 days following the moment of injection. Ultimately, the value of evaporation from the moment of injection until the 15th day after the injection of water, $E_{1-15}^{f_{2}}$, is described with the formula:

(9) $$E_{1-15}^{f_{2}}\left({\tilde{\theta }}_{1-5}\right)\;=3523.93\cdot {\tilde{\theta }}_{1-5}{}^{3.7559}$$

where: $E_{1-15}^{f_{2}}\left({\tilde{\theta }}_{1-5}\right)$—approximated function *f_2_* of evaporation for injection irrigation from the initial moment (1st day) until day 15 (mm), ${\tilde{\theta }}_{1-5}$—arithmetic mean value of weighted mean of volumetric moisture from the initial moment (1st day) until day 5 (cm^3^·cm^−3^), For the parameters a and b, selected as above, the value of the mean square from the differences $S_{f_{2}}^{1}$ (Equation (5)) was 1.82 mm^2^. The exponential function approximating $E_{1-15}^{f_{2}}\left({\tilde{\theta }}_{1-5}\right)$ is presented in Figure 12. The selected differences between $E_{1-15}$ and $E_{1-15}^{f_{2}}$ are also presented in Figure 12.

Studies on evaporation in soil monoliths were conducted by Tollenaar et al., who analysed changes in the structure of a loamy soil during drying [66]. One of the samples, with initial moisture of 0.34 cm^3^·cm^−3^, was dried at the temperature of 19.4 °C. After two days from the initial moment, the mass loss caused by evaporation amounted to 60 g. Referenced to the surface area of the sample (50.2 cm^2^), that decrease was 11.9 mm. Whereas, in the experiment under analysis in this paper, evaporation after two days, for a sample in the case of which a dose of 1000 cm^3^ of water was injected to the depth of 5 cm, amounted to 2.6 mm. A notably higher intensity of evaporation under laboratory conditions was obtained in a study by Śpitalniak et al. [67], in which experiments were conducted on a soil material with particle size distribution of its mineral parts corresponding to that of loamy sand, i.e., identical to that used in the experiment analysed herein. After two days, water loss relative to the surface of the sample was as much as 18.5 mm. However, it should be emphasised that in the course of the experiment, the sample was irrigated until the state of full saturation was achieved and heated with 110 W lamps. In the cited study, the soil samples were not irrigated with the injection method.

### 3.3. Evaluation of the Models $f_{1}\left(DI,HI\right)$ and $f_{2}\left(\tilde{\theta }\right)$

The goodness of values of $E_{1-15}$ approximated with functions $f_{1}\left(DI,HI\right)$ and $f_{2}\left(\tilde{\theta }\right)$ was estimated by comparing data obtained from calculations with data acquired on the basis of independent experiments. Equation (6) was used for the estimation of the goodness of approximation. Table 2 presents the values of $E_{1-15}$ obtained on the basis of the experiment with three pots, and values obtained on the basis of the functions $f_{1}\left(DI,HI\right)$ and $f_{2}\left(\tilde{\theta }\right)$. Also, this experiment confirmed the regularity proven earlier, that evaporation intensity decreases with increase in the depth to which water is supplied and increases with increase of the mean weighted volumetric moisture in the surface horizon of the soil. For example, with injection depth being 5 cm, the values of evaporation calculated with three methods are $E_{1-15}=4.79\;\mathrm{mm}$, $E_{1-15}^{f_{1}}=5.20\;\mathrm{mm}$, and $E_{1-15}^{f_{2}}=4.24\;\mathrm{mm}$ respectively, and for injection depth of 15 cm are much lower, at $E_{1-15}=1.33\;\mathrm{mm}$, $E_{1-15}^{f_{1}}=1.16\;\mathrm{mm}$, and $E_{1-15}^{f_{2}}=1.26\;\mathrm{mm}$. At the same time, when the mean weighted olumetric moisture is 0.167 cm^3^·cm^−3^, the evaporation is the same as for an injection depth of 5 cm, and when the mean weighted volumetric moisture is 0.121 cm^3^·cm^−3^, the evaporation value is lower and equal to that for an injection depth of 15 cm.

The value of the mean module of differences, $S_{f_{1}}^{2},$ for approximation on the basis of injection dose and depth was 0.63 mm. The value of the mean module of differences, $S_{f_{2}}^{2},$ for approximation on the basis of the weighted mean of volumetric moisture was 1.13 mm. This indicates that the function $f_{1}\left(DI,HI\right)$ permits greater precision of estimation of evaporation intensity of an injection-irrigated soil than the function $f_{2}\left(\tilde{\theta }\right)$. However, the method using TDR sensors for the estimation of evaporation intensity of an injection-irrigated soil has a greater potential for the construction of universal approximating models. This is due to the fact that the mean weighted volumetric moisture of the surface horizon is dependent on the kind of soil and on the parameters of injection irrigation. In addition, the advantage of the method based on the use of TDR sensors is that it uses arguments for the approximating function $f_{2}\left(\tilde{\theta }\right)$ in real time. Also taking into account the technical capabilities of the TDR set produced by the E-Test [44], it is possible to get arguments for the function $f_{2}\left(\tilde{\theta }\right)$ also in the field.

## 4. Conclusions

The paper presented two methods for the estimation of evaporation intensity of an injection-irrigated soil. In the first method, the input data are the parameters of injection irrigation, i.e., the dose of water and the depth of its application ($f_{1}\left(DI,HI\right)$). It was demonstrated that evaporation intensity increases with dose and decreases with the depth of water application. This relationship is approximated with a modified logistic function. Another presented method for the estimation of evaporation intensity of an injection-irrigated soil was a method in which the only data required for its application are the values of the weighted mean of volumetric moisture of the surface horizon of the soil $\left(\tilde{\theta }\right)$. It was demonstrated that evaporation intensity increases proportionally to the weighted mean of volumetric moisture. The study was conducted on a natural soil material, with particle size distribution of its mineral parts corresponding to that of loamy sand. The sole difference between this material and soil in its natural state was that it did not contain any organic parts and the fraction of particles larger than 2 mm. Comparison of the two methods indicated that the evaporation intensity of injection-irrigated soil can be estimated with higher accuracy when the domain of the approximating function is the injection depth and dose than in the case when the domain of the function is the weighted mean of volumetric moisture of the surface horizon of the soil. However, the method using TDR sensors for the estimation of evaporation intensity of an injection-irrigated soil has a greater potential for the construction of universal approximating models. In addition, the advantage of the method based on the use of TDR sensors is that it uses arguments for the approximating function $f_{2}\left(\tilde{\theta }\right)$ in real time.

## Figures and Tables

**Table 1:** Total evaporation from the moment of injection until day 15 ($E_{1-15}$) (mm).

Injection Depth (HI)	Water Dose (DI)			
	250 cm^3^	450 cm^3^	750 cm^3^	1000 cm^3^
5 cm	1.60	3.88	6.03	7.68
10 cm	1.40	2.37	4.30	6.82
15 cm	0.64	0.83	1.08	3.57

**Table 2:** Evaporation during initial 15 days for injection dose of 550 cm^3^ ($E_{1-15}$) calculated on the basis of the experiment with three pots, and on the basis of the functions $f_{1}\left(DI,HI\right)$ and $f_{2}\left(\tilde{\theta }\right)$ (Equations (8) and (9)).

Injection Depth (HI)	${\tilde{\theta }}_{1-5}$	$E_{1-15}$	$E_{1-15}^{f_{1}}$	$E_{1-15}^{f_{2}}$
(cm)	(cm^3^·cm^−3^)	(mm)	(mm)	(mm)
5 cm	16.7	4.79	5.20	4.24
10 cm	12.5	4.20	2.89	1.43
15 cm	12.1	1.33	1.16	1.26

## Data Availability

Not applicable.
